# Interpretable machine learning for endometriosis classification: a rule-based approach

**DOI:** 10.1186/s12911-026-03622-x

**Published:** 2026-06-10

**Authors:** Saeed Anbari Moghadam, Elham Akhondzadeh Noughabi, Shahideh Jahanian Sadatmahalleh

**Affiliations:** 1 Department of Industrial and Systems Engineering, Tarbiat Modares University, Tehran, Iran; 2 Department of Information Technology Engineering, Faculty of Industrial and Systems Engineering, Tarbiat Modares University, Tehran, Iran; 3 Department of Reproductive Health and Midwifery, Faculty of Medical Sciences, Tarbiat Modares University, Tehran, Iran

**Keywords:** Endometriosis, Rule-based model, CN2, Interpretability, Machine learning

## Abstract

**Background:**

Endometriosis is a chronic gynecological disease characterized by the growth of endometrial-like tissue outside the uterus, leading to pelvic pain, infertility, and other major health complications. Though some studies have tried to determine the factors that influence endometriosis and its clinical manifestations, the predictive modeling approaches have many limitations. Most of the models are based on complex algorithms that are not interpretable in a clinical setting, and therefore, practitioners cannot easily apply the findings. Moreover, much of the previous research often focuses on specific patient subsets or limited disease stages, leaving critical gaps in understanding the condition comprehensively. The purpose of this study was to address these challenges by using a rule-based model for analyzing various clinical data at each stage of endometriosis. The aim will be to develop interpretable and actionable diagnostic rules that will be able to empower health professionals in better understanding, diagnosis, and management of endometriosis while filling the existing gaps in knowledge within the field.

**Method:**

Clinical data were collected and preprocessed to deal with missing values, selection of relevant features, and standardization of variables. The CN2 rule induction algorithm was applied to 1,489 records with 52 clinical variables to generate interpretable classification rules linking clinical variables with the probability of endometriosis. The performance of the model was evaluated using several metrics: accuracy, F1-score precision, recall, and AUC. Derived rules were validated by clinical experts to ensure both statistical rigor and practical relevance.

**Result:**

The CN2 model achieved an AUC of 0.906 and a classification accuracy of 0.803 for binary classification. For multi-class classification of disease stages, the model demonstrated an average AUC of 0.705. Key findings were pelvic pain, dysmenorrhea, dyspareunia, severe bleeding, tumor markers, age, and BMI. The rules generated by the CN2 model provided interpretable insights, facilitating better understanding and management of endometriosis.

**Conclusion:**

The CN2 rule induction model has been effective in bridging the gaps in endometriosis research by providing interpretable and actionable diagnostic rules. Its integration into clinical practice may improve the precision of diagnosis, reduce diagnostic delays, and offer strategies for treatment, hence possibly improving patient outcomes.

**Clinical trial number:**

Not applicable.

## Introduction

Endometriosis is a chronic gynecological disease for which there are estimates that it affects approximately 190 million women worldwide [1, 2]. It is a non-malignant but often crippling condition thought to affect about 10% of the female population, based on studies of the general public reporting pelvic pain and subfertility [3]. Of the symptomatic patients, prevalence estimates are between 30% and 50% [4]. However, determining the true prevalence of endometriosis is problematic due to consistent underreporting, misdiagnosis, and cases that remain undiagnosed [1]. This disease has significant economic burden and poses a disease burden on society, but the full extent of its impact remains unknown [5]. Endometriosis is characterized by the abnormal growth of tissue similar to the endometrium in locations other than the uterus [6], typically involving the ovaries, bowel, bladder, and peritoneum [7]. Endometriosis was staged using the revised American Fertility Society (r-AFS) system, which divides the extent of the disease into four stages: stage 1-minimal, stage 2-mild, stage 3-moderate, and stage 4-severe [8]. The symptoms and signs of endometriosis are often non-specific and vary in severity, further contributing to the clinical heterogeneity of the disease and making its diagnosis challenging [9]. Moreover, various factors make diagnosing endometriosis problematic, including a broad differential diagnosis, lack of awareness, unnecessary diagnostic investigations, and lack of reliable non-invasive diagnostic tools [1, 10–12]. Consequently, many patients are significantly delayed from receiving a conclusive diagnosis; literature reports this delay in diagnosis as anywhere from 6 to 12 years worldwide [1, 13, 14].

In the past years, data mining practices have been increasingly employed in the clinical domain to analyze various types of data, including clinical datasets [15]. So far, these methods have found applications in various clinical applications: decision-making, patient self-management approaches, unraveling the cause of a particular disease, and drug development studies [15, 16]. However, their use in the context of endometriosis study is usually limited because these algorithms are mostly complex and require expert involvement for proper interpretation and model development [15]. Considering the complex characteristics of endometriosis and the capabilities of machine learning algorithms to tackle these intricacies, a significant opportunity arises to utilize these methodologies for enhancing non-invasive diagnostic procedures, which may lead to a decrease in diagnostic delays and a reduction in human error [17].

Notwithstanding the potential of machine learning to further the research on endometriosis, a notable constraint persists regarding the interpretability of various current predictive models. Most of the models rely on complex algorithms that are challenging for clinicians to interpret and translate into clinical practice [18]. Moreover, the majority of existing research [6, 19–22], tends to have a limited scope, often focusing exclusively on certain patient demographics or specific stages of the disease, thereby failing to provide a holistic evaluation of case populations throughout all stages of endometriosis. In the course of previous studies, the objective of this research is to employ a rule-based algorithm to identify critical determinants that affect endometriosis and to explore the interconnections among these factors, including a broad spectrum of clinical variables. Rule-based systems constitute expert models that employ a collection of “If-then” rules generated from both observed patterns and specialized knowledge, rendering them especially appropriate for intricate tasks including classification, regression, association, and prediction [23]. This research contributes by implementing a rule-based methodology for analyzing endometriosis across both control and case populations, considering all the stages of the disease. The approach offers more complete insight into the factors contributing to endometriosis, thereby enhancing diagnostic practices due to interpretable rule generation in a more holistic way. Such rules will make clinical decisions more perspicacious, providing more distinct insights for health professionals. This ultimately ensures a better diagnostic precision and efficiency in the management of endometriosis at any stage of the disease and leads to better patient care.

## Related works

Many machine learning approaches have been used to analyze clinical data and provide wide-ranging information about endometriosis and predictions of its appearance [19–21, 24, 25]. One study introduced a nomogram developed by multivariate logistic regression analysis to predict postoperative voiding dysfunction in women undergoing surgery for deep endometriosis. Clinical and surgical variables included age, colorectal involvement, colpectomy, and parametrectomy in model development [26]. Another study focused on the application of advanced machine learning techniques, namely Logistic Regression and Gradient Boosting, in order to predict the occurrence of endometriosis based on a patient’s medical history. The models identified key diagnostic and procedural codes with a possible allowance for early prediction of the disease. The results demonstrate the potential of machine learning algorithms in predicting the onset of endometriosis and hence assist in early medical interventions and improving patients’ outcomes [25]. Similarly, one study implemented machine learning algorithms such as logistic regression, decision tree, random forest, and Gradient Boosting in the diagnosis of endometriosis, identifying 16 critical features that included clinical and symptoms-related information, which had promising accuracy in identifying cases of endometriosis [24]. A recent retrospective study assessed the usefulness of machine learning for the diagnosis of endometriosis from ultrasonographic characteristics. On a dataset of 505 patients (149 confirmed cases), the authors trained and tested multiple ML algorithms using stratified 5-fold cross-validation. The top-performing algorithms were Support Vector Machine, Random Forest, Extra-Trees, and Gradient Boosting [27]. Seven machine learning algorithms were used in a study to predict the risk of stage 4 endometriosis in a cohort of 308 patients. Random Forest had the highest discriminative power, performing better than the other models [22]. Another example of using machine learning techniques is a predictive study of endometriosis based on self-reported symptoms. Using questionnaire data from both women with and without endometriosis, researchers trained models using techniques like Decision Tree, Random Forest, Gradient Boosting Classifier, and Adaptive Boosting [20]. Similarly, another study used a 21-item patient questionnaire with pain and infertility symptoms, using logistic regression model [28]. Another study analyzed a substantially larger cohort of 148,647 women to distinguish those with and without an endometriosis diagnosis. Leveraging over 1,000 variables, including genetic data, medical history, and lifestyle factors. The researchers employed gradient boosting algorithms for this study [29].

Previous studies have provided useful insights into the factors affecting endometriosis and clinical features of patients suffering from it. However, several drawbacks are still prominent. One of the key limitations involves the interpretability associated with most predictive models; these are often based on complex algorithms that prevent clinicians from gaining useful insights. Second, there is a lack of rule-based models applied to various clinical data, which can give clearer and more interpretable paths in decision-making at different stages of endometriosis. Another limitation involves focusing only on narrow patient groups or selected disease stages. Most studies lack comprehensive coverage that should involve both control and case populations and address all disease stages of endometriosis.

## Research method

### Methodology overview

**Fig. 1:**
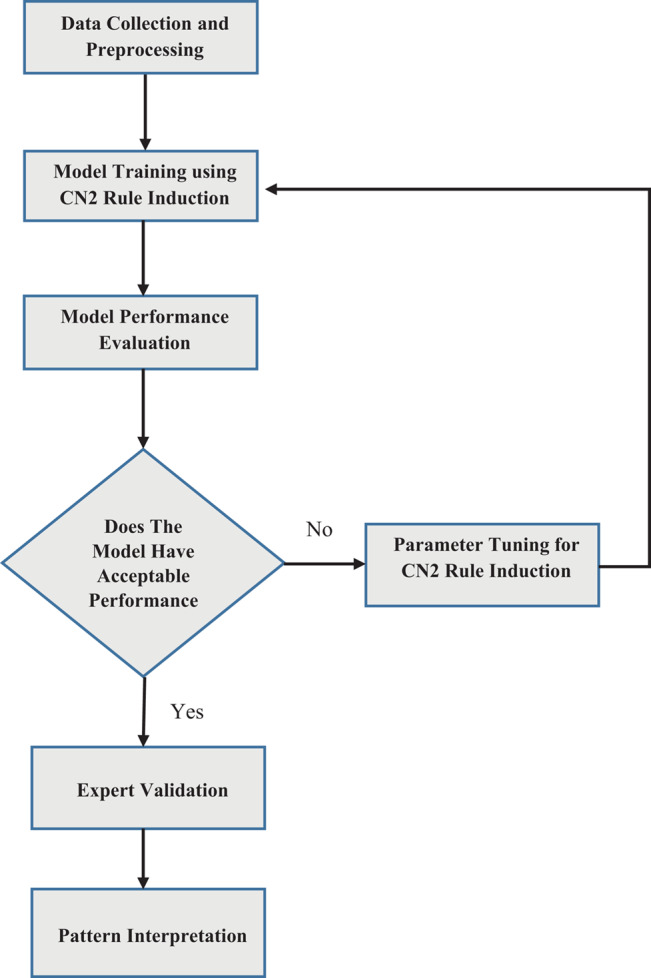
Research methodology to identify influential factors as rules in endometriosis

The methodology to be followed for this research involves a number of successive steps that ensure the results are robust and interpretable. First is the data collection and preprocessing of the data, which describes the process by which raw data is cleaned and transformed to best prepare it for analysis. This generally includes handling missing values, selecting relevant features, and normalization or standardization, where appropriate. After preprocessing, the preprocessed data is used as input to the CN2 rule induction algorithm. CN2 is a classifier that operates based on predefined rules, distinguished by its clarity and user-friendliness in medical settings. The algorithm produces straightforward and transparent rules that elucidate the connections between clinical variables and the probability of endometriosis. For example, it may uncover a rule such as: “If a patient is experiencing pelvic pain and is over age 34 then she is likely to be suffering from endometriosis”. Each of these individual rules is very helpful for clinicians and researchers in order to understand and communicate the related conditions of endometriosis [30]. Once the CN2 model is trained, the next step involves model testing or evaluation. The precision, recall, accuracy, and other performance measures of the model are then strictly scrutinized. If the model meets the developed performance criteria, the set of rules generated by CN2 is then passed on to clinical experts for validation. This expert validation is a very important step because, in this manner, validity is established both from a statistical standpoint and from a clinical one, ensuring findings that truly add to our understanding of the determinants of endometriosis. If the model performance is not satisfactory, we proceed to carry out a parameter tuning step. During this step, several of the CN2 algorithm parameters are varied in pursuit of improved accuracy of the model. We then retrain our model after tuning and repeat the step of performance evaluation until the desired results are obtained. In this way, the iterative process ensures that the final model will be accurate and interpretable, a very important tool in the understanding and diagnosis of endometriosis. Figure 1 presents the research methodology process. Besides, the authors provide a comparative analysis between the proposed technique and other classifiers.

### Data collection and preprocessing

In this research, we utilized an endometriosis dataset collected over a span of 10 years by the Royan Institute for Reproductive Biomedicine. To get the data ready for analysis, a number of preprocessing steps were taken. First, the original endometriosis data were divided across three separate datasets. We merged these datasets, consolidating all common variables into a single comprehensive dataset. The dataset contained 1500 records and 79 variables describing control and case patients. Second, following the guidelines of [31], we removed columns and rows with more than 60% missing values. For instance, variables such as “age of pregnancy” (with 79% missing values), “age at marriage” (with 95% missing values), and “delivery type” (with 90% missing values) were excluded. Skewness and kurtosis were then computed for numeric variables to check for their distributional properties. Age, Weight, Height, BMI, and Menarche had skewness values near zero and Pearson kurtosis values near 3, indicating approximately normal distributions, whereas Length of menstrual cycle was moderately right-skewed (skewness = 1.04, kurtosis = 3.17). In contrast, the number of Ectopic pregnancies was extremely skewed (skewness = 9.22, kurtosis = 94.23). Initially, we removed this variable due to its very low variance; however, the performance of the ML models slightly decreased. Therefore, despite its skewness, the variable carried predictive information, and we retained it along with other clinically significant but skewed variables. Some clinically significant variables, including the number of pregnancies and abortions, were kept despite skewness, following advice from domain experts, since they remain relevant for clinical interpretation and can be effectively handled by non-parametric machine learning models. Because of the clinical nature of the data, we anticipated skewed distributions that were considered informative. Finally, feature selection was undertaken with the help of experts to ensure the dataset consisted solely of clinical variables; dietary, post-surgery information, and other non-clinical variables were removed. Following preprocessing, the resulting dataset consisted of 1,489 records and 52 variables that described the features of both control (700 samples) and case (789 samples) patients. Staging within the case group included 162 patients at stage 1, 179 at stage 2, 237 at stage 3, and 213 at stage 4. A detailed description of the variables used in this research and key demographic and clinical characteristics can be found in Tables 1 and 2.

**Table 1:** Description of variables used in the research

Variable Name	Description
Case control	Indicates whether the case is a control or a study case (endometriosis or no endometriosis).
Endometriosis Stage	The stage of endometriosis diagnosed in the patient (e.g., Stage 1, 2, 3, 4).
Age	The age of the patient in years.
Job	The occupation of the patient, e.g., Housewife.
Weight	The weight of the patient in kilograms.
Height	The height of the patient in centimeters.
Sport	Indicates whether the patient engages in sports (physical activity).
BMI	Body Mass Index, calculated from weight and height.
Type of infertility	Type of infertility the patient has (e.g., primary, secondary).
Live birth	Indicates whether the patient has had a live birth.
Length of menstrual cycle	The length of the menstrual cycle in days.
IUD	Indicates whether the patient uses an Intrauterine Device.
Duration of infertility	The duration of infertility in years.
Menarche	The age at which the patient had her first menstruation.
Menstrual Status	The regularity of the patient’s menstrual cycle (e.g., regular, irregular).
Duration of bleeding menstrual	The duration of menstrual bleeding in days.
Number of pregnancy	The number of pregnancies the patient has had.
Gravidity	Indicates the number of times a woman has been pregnant.
IUFD	Indicates whether the patient has experienced Intrauterine Fetal Death.
Ectopic pregnancy	Indicates whether the patient has had an ectopic pregnancy.
Number of EP	The number of ectopic pregnancies the patient has had.
Number of delivery	The number of deliveries the patient has had.
History of previous abortion	Indicates whether the patient has had a previous abortion.
Number of abortion	The number of abortions the patient has had.
Smoking	Indicates whether the patient smokes.
Family history risk of endometriosis	Family history of risk factors for endometriosis.
History of Galactorrhea	History of galactorrhea (spontaneous milk flow).
Abnormally of genital	Indicates the presence of genital abnormalities.
History of STD	History of sexually transmitted diseases (STDs).
History of PID	History of pelvic inflammatory disease (PID).
Dysmenorrhea	Presence of painful menstruation.
Dyspareunia	Presence of painful intercourse.
Total.Pelvic.Pain	The overall presence of pelvic pain.
Pelvic.pain	Specific details about pelvic pain.
PelvicAndLowerBackPain	Pain in the pelvic and lower back area.
L. endometriosis symptoms	describe symptoms related to endometriosis
Inguinal Pain	Pain in the groin area.
Severe bleeding	Indicates the presence of severe menstrual bleeding.
Irregular bleeding	Indicates the presence of irregular menstrual bleeding.
Premenstrual Spotting	Indicates the presence of premenstrual spotting.
Fatigue	Indicates the presence of fatigue symptoms.
Diarrhea	Indicates the presence of diarrhea symptoms.
Constipation	Indicates the presence of constipation symptoms.
Bloat-Nausea-Vomiting	Indicates the presence of bloating, nausea, or vomiting.
Dyschezia	Indicates the presence of difficulty in defecation.
Dysuria	Indicates the presence of painful urination (dysuria).
Tumor.marker.CA	Indicates the presence of CA marker (positive/negative).
History of Pelvic Surgery	History of previous pelvic surgery.
Previous Cervical Trauma	Indicates whether the patient has had previous cervical trauma.
Dysmenorrhea_dyspareunia_pelvic_pain	The combined presence of dysmenorrhea, dyspareunia, and pelvic pain.
Dysmenorrhea_Dyspareunia	Combined assessment of dysmenorrhea and dyspareunia.
Dysmenorrhea_Total.Pelvic.Pain	Combined assessment of dysmenorrhea and total pelvic pain.
Dyspareunia_Total.Pelvic.Pain	Combined assessment of dyspareunia and total pelvic pain.

**Table 2:** Key demographic and clinical characteristics of the study cohort

Variable	Value
Total patients	1489
Cases	789
Controls	700
**Continuous variables (mean ± SD)**	
Age (years)	31.33 ± 5.19
BMI (kg/m²)	25.29 ± 3.98
Menarche (years)	13.23 ± 1.36
Length of menstrual cycle (days)	29.39 ± 4.50
Duration of infertility (years)	6.97 ± 4.57
Duration of menstrual bleeding (days)	6.07 ± 1.83
**Categorical variables n (%)**	
Dysmenorrhea (yes)	991 (66.6%)
Family history of endometriosis (yes)	69 (4.6%)
Dyspareunia (yes)	490 (32.9%)
Premenstrual spotting (yes)	261 (24.0%)
Dyschezia (yes)	121 (11.1%)
Job (Housewife)	1202 (80.7%)
Pelvic pain (yes)	249 (22.9%)
Severe bleeding (yes)	193 (17.8%)
Positive tumor marker CA	254 (38.7%)
**Disease stage (r-AFS classification)**	
Stage 1	162
Stage 2	179
Stage 3	237
Stage 4	213

### Model training using CN2 rule induction

The CN2 algorithm, as developed by Peter Clark and Tim Niblett in the early 1980s, is a rule-based classification procedure that is used widely in research in data mining and knowledge discovery domains [32]. Being a covering algorithm, CN2 is capable of generating indicative rules of classification from labeled datasets and is thus well suited to categorical data applications. This ability to generate rules understandable by humans, in turn, is of special value in areas like healthcare, where transparency of decision-making processes cannot be overlooked. Besides that critical advantage, CN2 also possesses the capability to handle both continuous and categorical variables for any given dataset. It does so by discretizing the continuous features into intervals; hence, the algorithm outputs the rules based on these discretized intervals. Such flexibility would guarantee that CN2 is applicable to any dataset, covering multiple datatypes without much preprocessing effort. It is also very robust to noisy data and outliers. It is designed to run effectively even when the training data is incomplete or faulty [33]. Following a principle, it incorporates statistical measures like information gain in rule evaluation and optimization that reduce the impact of irrelevant attributes on model performance. This pruning process helps to mitigate overfitting and ensures that the generated rules remain both concise and effective. Another salient point of the CN2 algorithm is its use of beam search toward rule generation, generating large numbers of rule hypotheses, and then selecting the best under pre-defined conditions. However, the methodology allows the algorithm to detect subtle patterns in the data while keeping a good balance between the strength of the rules and their simplicity. CN2, then, has gained a reputation for its scalability as it can develop understandable, intelligible rule sets which is of special benefit in domains where the understanding of intrinsic reasoning is as important as the forecast itself. Beyond other things, the strengths of CN2 are interpretability, scalability, and robustness to noise, as one of the best options to induce rule-based classifiers when comprehensibility and transparency are of crucial relevance [34]. This section is an outline pseudocode on the working of the CN2 algorithm:

1. Initial Setup: Begin with an empty rule list and a data set that consists of instances with corresponding class labels.
2. Rule Generation: Iteratively generate rules to classify instances by selecting conditions (conjunctions of attribute-value pairs) that maximize information gain or another relevant metric. These conditions form the basis of the rule.
3. Rule Evaluation: The rule that has been met will be evaluated for its performance in terms of accuracy on the target instances in the training data.
4. Pruning Rules: Eliminates or modifies inconsequential rules that account for very little increase in classification accuracy and cause overfitting.
5. Procedure Iteration: Steps 2–4 are repeatedly followed unless the classification accuracy of the problem is no longer improved or a stopping criterion is satisfied.
6. Building Final Rules: It generates a list of rules described by a set of conditions that once met predict the class label of an instance [30].

### Model performance evaluation

The performance comparison of the CN2 rule induction algorithm has been performed using the following metrics: accuracy, sensitivity, specificity, precision, recall, and ROC curve. In a way, the calculations of accuracy, sensitivity, and specificity are materialized by means of elements, such as True Positive, True Negative, False Positive, and False Negative, emanating from the confusion matrix. The sample from a “False Positive-FP” is the type that does not really come under the positive class but is wrongly predicted as being so. “True Positive-TP” is a sample for which the prediction becomes correct for the positive class. “False Negative-FN” refers to those types of instances that have been mispredicted as negative when they truly belong to the positive class. Finally, “True Negative (TN)” is the sample that is correctly classified as part of the negative class instance [35]. The section below describes the metrics that will be used to quantify the predictive performance of the developed models:

1 $$\:Accuracy=\frac{TP+TN}{TP+TN+FP+FN}$$

2 $$\:Recall=\frac{TP}{TP+FN}$$

3 $$\:Precision=\frac{TP}{TP+FP}$$

4 $$\:F1\:Score=2\times\:\left(\frac{1}{\frac{1}{Precision}+\frac{1}{Recall}}\right)$$

5 $$\eqalign{ & MCC \cr & = {{(TP \times TN) - (FP \times FN)} \over {\sqrt {(TP + FP) \times (TP + FN) \times (TN + FP) \times (TN + FN)} }} \cr}$$

### Parameter tuning for CN2 rule induction

Optimization of the CN2 rule induction algorithm is a fine-tuning process of its parameters toward higher performance-quality models of induced rules. Though CN2 possesses fewer parameters compared to most machine learning algorithms, a few crucial tuning parameters do make a vast difference in the way it performs. It is appropriate to note that the important parameters to be tuned in CN2 are: Beam width-it expresses the number of rule hypotheses considered at each search step. Larger beam widths allow alternative hypotheses to be followed up but at the cost of increased computational complexity. The minimum coverage threshold determines the smallest number of instances a rule should cover when expanded. Lower values will make the rules more specific, while higher values will make them more general and less interpretable. The minimum significance threshold determines the minimal improvement in predictive accuracy for a rule to be considered significant; lower thresholds may lead to more rules, whereas higher thresholds result in fewer but more accurate rules, so a balance between the quantity and quality of rules needs to be found. Techniques for post-pruning, such as rule covering and rule trimming, eliminate superfluous or redundant rules from the rule set, thus enhancing model performance and interpretability. Therefore, in this way, an imbalanced dataset tends to tune its parameters by adding a minimum coverage threshold or class weights and deals with problems related to class imbalance, thus increasing model accuracy. Cross validation should be chosen such that it is either k-fold or stratified cross-validation, so that the modeling performance is reliable and able to generalize across more dissimilar data subset splits. Although CN2 requires fewer parameter adjustments than some other machine learning techniques, careful tuning of these parameters is likely to influence the generated rules’ accuracy and interpretability strongly. In most cases, parameters essentially require continuous experimentation and refinement to work out an optimal setting for some particular dataset and task [34].

### Expert validation

Expert validation of this research was part of the methodology for analyzing and refining rules extracted from the CN2 model. More precisely, the outputs of the CN2 model, consisting of case-control classification rules and staging rules, were presented to an expert in gynecology and endometriosis studies. In that respect, collaboration ensured a comprehensive understanding of the influential factors identified by the model and their applicability to clinical practice.

### Pattern interpretation

The interpretability of the CN2 algorithm results in simple and easy-to-understand rules. These rules can be well comprehended and evaluated even by stakeholders such as clinicians and researchers. The rules point out specific conditions or a combination of factors relevant to classifying endometriosis cases. This also creates room for transparency, whereby experts can check the clinical relevance and plausibility of the identified pattern and make sure that the final rule set meets current medical knowledge and diagnostic considerations in practice.

## Results

**Fig. 2:**
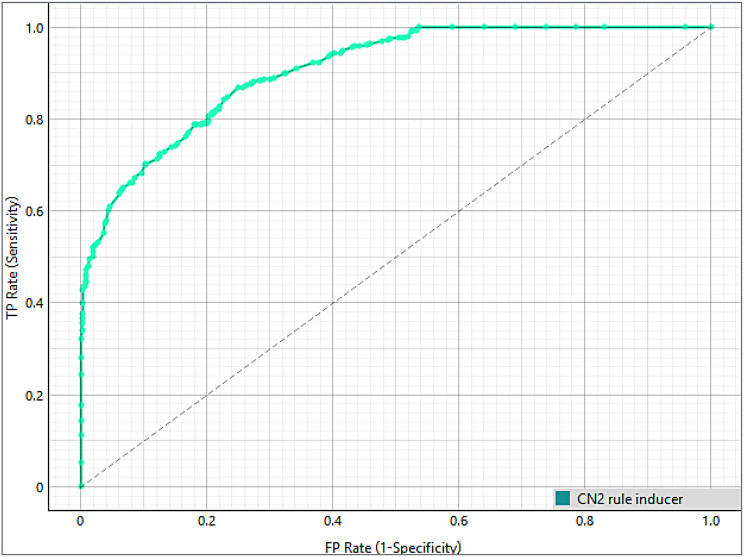
CN2 inducer ROC for case

**Fig. 3:**
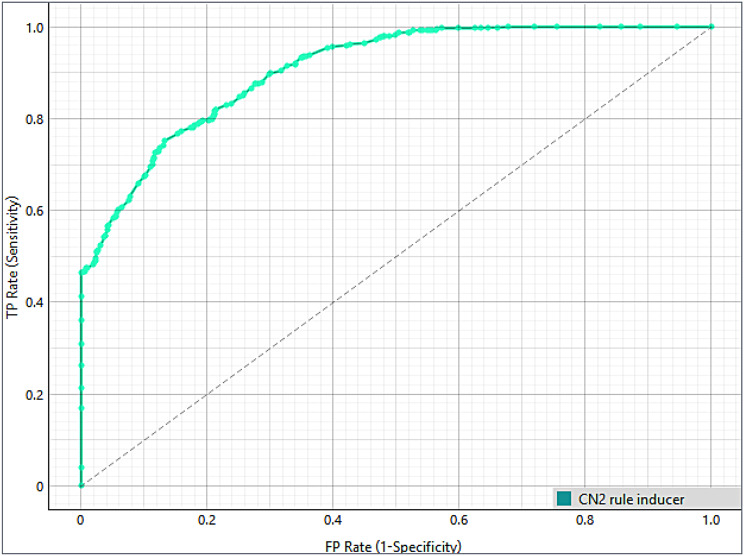
CN2 inducer ROC for control

**Fig. 4:**
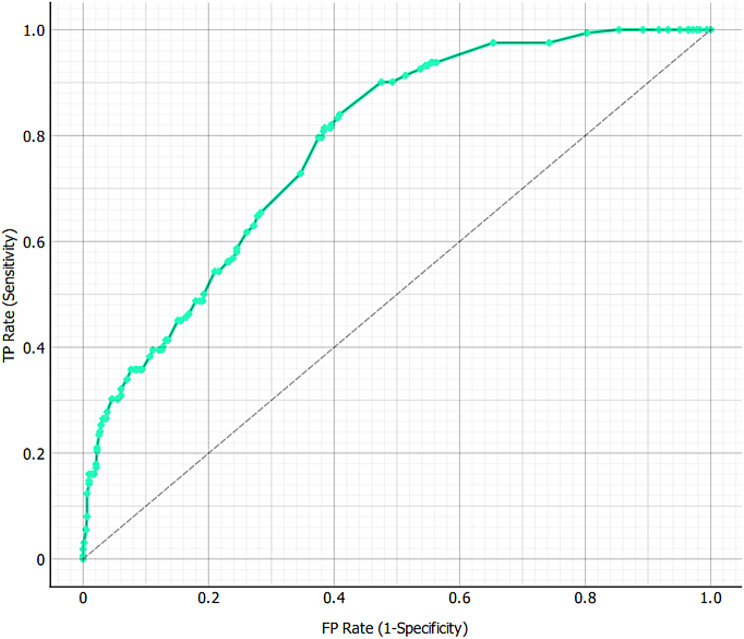
CN2 inducer ROC for stage 1

**Fig. 5:**
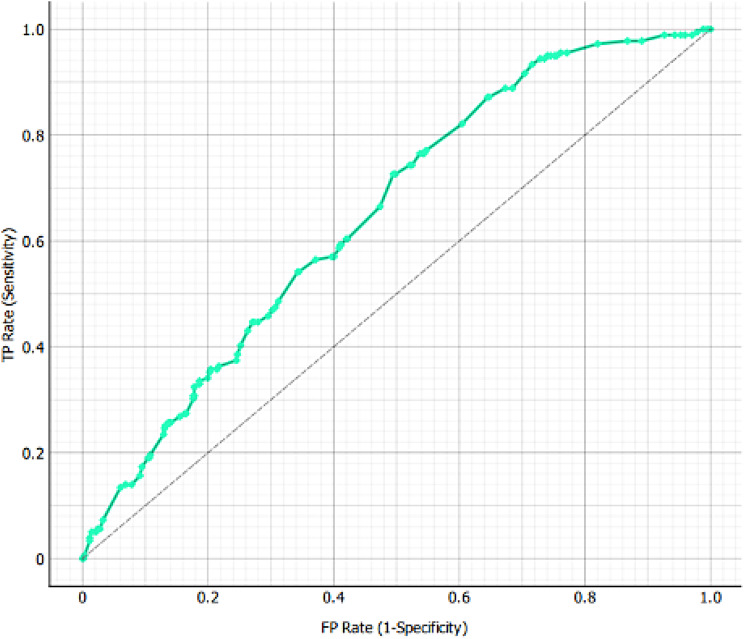
CN2 inducer ROC for stage 2

**Fig. 6:**
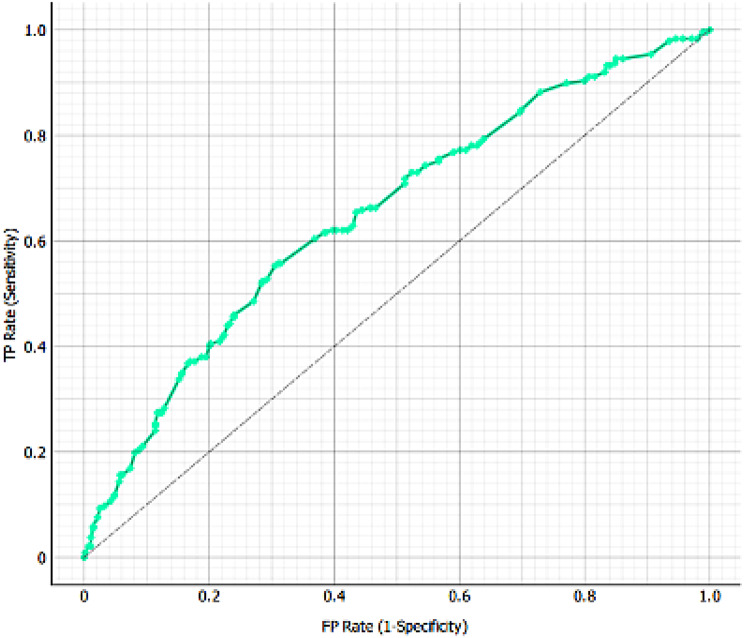
CN2 inducer ROC for stage 3

**Fig. 7:**
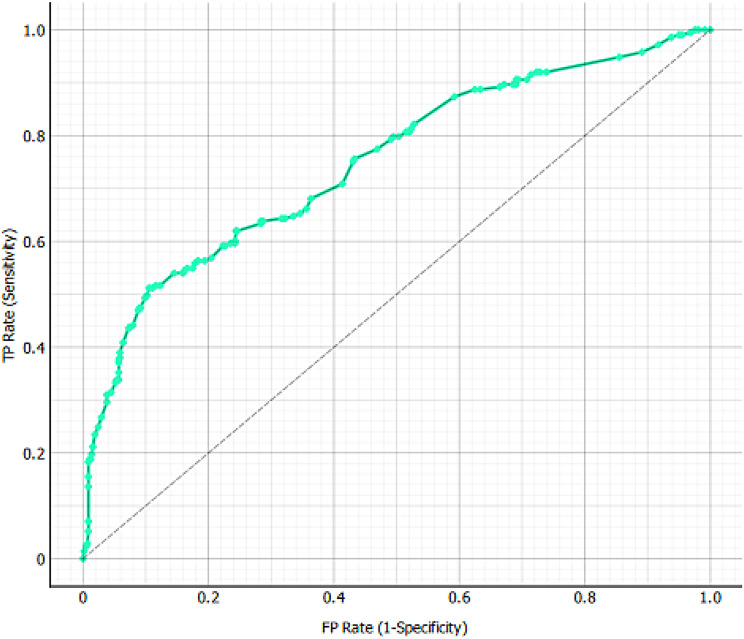
CN2 inducer ROC for stage 4

We used a rule-based approach to analyze the major influential factors for endometriosis identification. We developed a rule set using the CN2 algorithm, initially generating a set of rules that was subsequently refined through expert validation. After validation, we finalized 29 rules for case and control classification listed in Tables 3 and 25 rules for staging endometriosis from stages 1 to 4 listed in Table 4. Following were the conditions set to yield the rules: ordering in the rule set: ordered; covering algorithm: exclusive; evaluation measure: Laplace accuracy; beam width: 5; minimum coverage of a rule: 15; maximum length of a rule: 3; default alpha: 1.0, parent alpha: 1.0. These rules captured complex dependencies between factors. Such rules could be derived, tested, and refined iteratively by CN2 rules, therefore outlining the data’s hidden patterns. To assess our findings in terms of the effectiveness of our rule-based predictor of key endometriosis factors, we performed stratified 10-fold cross-validation where in each fold approximately 90% of the data were used for training and 10% for testing. The Receiver Operating Characteristic (ROC) curve for the target class (control, case), presented in Figs. 2 and 3, showed an average area under the curve (AUC) of approximately 0.90. Additionally, the ROC curve for the target class (stages 1–4), depicted in Figs. 4, 5 and 6, and 7 showed a fair accuracy level. An average AUC of about 0.70 for the four stages. The experiments were conducted using the Python-based software ‘Orange (v3.37)’ [36].

Table 3 defines the condition and classification (Case or Control) category, the distribution of instances in each category, and the length of the rule. Nineteen rules out of the twenty-nine induced are for the case class, whereas the remainder, according to Table 3 below, are associated with the control class. Of these, the following rules were contributing key features to the classification as a case of endometriosis that made the case class dominant: Pelvic and lower back pains, Severe bleeding, Positive tumor marker CA results, BMI, Age, Total Pelvic pain, Dyspareunia, and Dysmenorrhea. Some of the important rules leading to case classification are as follows:

- Rule 1: A positive tumor marker CA categorizes the individual as a case.
- Rule 2: The presence of pelvic and lower back pain without irregular menstrual status suggests a case classification.
- Rule 3: Total pelvic pain, coupled with an age of 31 or older, is a predictor for being classified as a case.
- Rule 4: Severe bleeding beginning at the age of 13 and after 13, increases the likelihood of a case classification.

However, the control class was dominating where: factors such as the absence of dysmenorrhea, and higher BMI were found to be key indicators for a control classification.

**Table 3:** Induced rules from CN2 model for target class (Control, Case)

#	IF Conditions	THEN Class	Distribution [# of Case, # of Control]	Laplace Estimate
1	Tumor.marker.CA=positive	Case	[254, 0]	0.996
2	PelvicAndLowerBackPain = yes AND Menstrual Status≠irregular	Case	[83, 0]	0.988
3	Total.Pelvic.Pain = Yes AND age ≥ 31.0	Case	[34, 0]	0.972
4	Severe bleeding = yes AND Menarche ≥ 13.0	Case	[20, 0]	0.955
5	Irregular bleeding = yes AND PelvicAndLowerBackPain = no AND Menarche ≥ 12.0	Case	[17, 0]	0.947
6	Total.Pelvic.Pain = Yes AND BMI ≤ 24.49 AND Duration of bleeding menstrual ≥ 5.0	Case	[15, 0]	0.941
7	Family history risk of endometriosis = yes AND BMI ≤ 23.14	Case	[15, 1]	0.889
8	Length of menstrual cycle ≥ 31.0 AND Weight ≥ 76.0 AND Premenstrual Spotting = no	Control	[1, 15]	0.889
9	Length of menstrual cycle ≥ 31.0 AND Duration of infertility ≥ 8.0 AND Duration of bleeding menstrual ≥ 7.0	Control	[1, 17]	0.900
10	Length of menstrual cycle ≥ 34.0 AND Dysmenorrhea = no	Control	[1, 17]	0.900
11	Premenstrual Spotting = yes AND Dysmenorrhea = no AND Family history risk of endometriosis = no	Case	[15, 1]	0.889
12	Dysmenorrhea = no AND BMI ≥ 29.76 AND Height ≥ 154.0	Control	[0, 22]	0.958
13	Dysmenorrhea_Dyspareunia = yes AND Duration of infertility ≤ 6.0 AND Age ≥ 31.0	Case	[30, 3]	0.886
14	Number of abortion ≥ 2.0 AND Duration of bleeding menstrual ≤ 6.0	Control	[3, 13]	0.778
15	BMI ≤ 22.95 AND Sport = yes AND Weight ≥ 58.0	Case	[15, 0]	0.941
16	BMI ≤ 22.35 AND Job≠House Wife AND Length of menstrual cycle ≥ 27.0	Case	[19, 1]	0.909
17	Dyspareunia = yes AND Previous Cervical Trauma = yes AND Job=House Wife	Case	[14, 2]	0.833
18	Dysmenorrhea = no AND Age ≤ 25.0 AND Height ≥ 157.0	Control	[1, 14]	0.882
19	Duration of infertility ≥ 8.0 AND History of Galactorrhea = no AND Menarche ≤ 14.0	Control	[4, 35]	0.878
20	Weight ≤ 54.0 AND Dysmenorrhea = yes AND Age ≥ 30.0	Case	[16, 2]	0.850
21	Dyspareunia = yes AND Weight ≤ 62.0 AND Duration of infertility ≥ 3.0	Case	[28, 7]	0.784
22	Live birth = Yes AND BMI ≥ 25.0 AND Job=House Wife	Control	[2, 13]	0.824
23	Sport = yes AND Dyspareunia = no AND Height ≥ 159.0	Case	[17, 3]	0.818
24	Dysmenorrhea = no AND Length of menstrual cycle ≥ 29.0 AND BMI ≥ 22.35	Control	[4, 21]	0.815
25	Duration of infertility ≤ 1.5 AND Length of menstrual cycle ≥ 25.0	Case	[12, 4]	0.722
26	BMI ≥ 27.06 AND Weight ≤ 70.0 AND Length of menstrual cycle ≥ 23.0	Case	[14, 2]	0.833
27	Age ≥ 34.0 AND Severe bleeding = yes AND Length of menstrual cycle ≥ 28.0	Case	[12, 3]	0.765
28	Duration of bleeding menstrual ≤ 5.0 AND Weight ≥ 60.0 AND Family history risk of endometriosis = no	Control	[4, 11]	0.706
29	Age ≥ 28.0 AND Bloat-Nausea-Vomiting = yes AND BMI ≥ 21.48	Case	[15, 2]	0.842
30	TRUE	Case	[789, 700]	0.530

Table 4 focuses on the significant variables influencing the development of endometriosis, stages 1 through 4. The Endometriosis was staged according to the revised American Fertility Society classification (r-AFS), which classifies the disease as stage 1-minimal, stage 2-mild, stage 3-moderate, and stage 4-severe [8]. In this first stage of endometriosis, the criteria are tied to a combination of physiologic and symptomatic features that help in defining early stage endometriosis. Some notable rules leading to stage 1 classification include:

- Rule 6: Indicates a negative tumor marker CA and a BMI of 25.4 or higher, suggests an early-stage of endometriosis.
- Rule 12: The presence of previous cervical trauma, absence of dysmenorrhea, and engagement in sports activities are linked to stage 1 classification.

Stage 1, underlines the features of BMI, the presence of pelvic pain, and lifestyle factors such as physical activity affecting the initial stage of the disease. Stage 2 classification is related to some more specific nature of symptoms along with patient history indicators, usually from the categories of gastrointestinal and reproductive health. Some of the major rules that lead to stage 2 classification are:

- Rule 9: The presence of constipation and a BMI of 23.44 or lower, without fatigue, is an indicator of stage 2 endometriosis.
- Rule 14: A history of pregnancy, infertility duration of three or more years, and a BMI of 19.81 or higher are influential factors pointing towards stage 2.
- Rule 17: The presence of genital abnormality, experience menstrual cycles lasting 28 days or more, and weigh 66 kg or less. helps classify a patient in stage 2.

These rules highlight gastrointestinal symptoms, including constipation, and reproductive history including pregnancy and infertility as important aspects of disease development toward stage 2. The stage 3 classification rules express a progression in the severity and combination of symptoms. The influential rules for the classification for stage 3 include:

- Rule 5: Patients showing symptoms of endometriosis lesions and endometrioma cysts, coupled with inguinal pain, and no presence of dysuria are likely to be classified as stage 3.
- Rule 8: Indicates that a family history of endometriosis, body weight over 59 kg, and a menstrual cycle of 29 days or less are suggestive of stage 3.

Factors influencing stage 3 classification depend primarily on symptoms involved, family medical history, a positive tumor marker CA, and parameters of reproductive health. Stage 4, representing the most severe form of endometriosis, is characterized by complex combinations of symptoms and patient history. Some significant rules for stage 4 classification include:

- Rule 2: The presence of a positive tumor marker CA, a history of infertility lasting 12 or more years, and symptoms of endometriosis lesions indicate stage 4 endometriosis.
- Rule 3: Combines a positive tumor marker CA, engagement in sports (physical activity), and absence of pelvic or lower back pain as factors contributing to a stage 4 classification.
- Rule 4: The presence of both endometriosis lesions and endometrioma cysts, history of pelvic surgery, and being a housewife further indicate stage 4.

Stage 4 is the most serious and carries a positive tumor marker, prolonged infertility, and related complex symptomatology with several symptoms occurring simultaneously.

**Table 4:** Induced rules from CN2 model for target class (Stages 1–4)

#	IF Conditions	THEN Class	Stage Distribution [Stage1, Stage2, Stage3, Stage4]	Laplace Estimate
1	L.endometriosis symptoms=endometrioma.cysts AND Sport = no AND BMI ≤ 26.56	Endometriosis.Stage=Stage4	[0, 0, 1, 20]	0.840
2	Tumor.marker.CA=positive AND Duration of infertility ≥ 12.0 AND L.endometriosis symptoms=endometriosis.lesions	Endometriosis.Stage=Stage4	[0, 0, 1, 19]	0.833
3	Tumor.marker.CA=positive AND Sport = yes AND PelvicAndLowerBackPainC = no	Endometriosis.Stage=Stage4	[0, 0, 7, 32]	0.767
4	L.endometriosisSymptoms=endometriosis.lesions&endometrioma.cysts AND History of Pelvic Surgery = Yes AND Job=House Wife	Endometriosis.Stage=Stage4	[0, 1, 2, 16]	0.739
5	L.endometriosisSymptoms=endometriosis.lesions&endometrioma.cysts AND Inguinal Pain = yes AND Dysuria = no	Endometriosis.Stage=Stage3	[0, 0, 15, 1]	0.800
6	Tumor.marker.CA=negative AND BMI ≥ 25.4	Endometriosis.Stage=Stage1	[22, 2, 0, 0]	0.821
7	Tumor.marker.CA=positive AND Length of menstrual cycle ≥ 31.0 AND Dysmenorrhea = yes	Endometriosis.Stage=Stage4	[0, 0, 2, 13]	0.737
8	Family history risk of endometriosis = yes AND Weight ≥ 59.0 AND Length of menstrual cycle ≤ 29.0	Endometriosis.Stage=Stage3	[1, 1, 13, 1]	0.700
9	Constipation = yes AND BMI ≤ 23.44 AND Fatigue = no	Endometriosis.Stage=Stage2	[0, 12, 3, 1]	0.650
10	Tumor.marker.CA=positive AND Number of pregnancy ≥ 1.0 AND Diarrhea = no	Endometriosis.Stage=Stage3	[0, 0, 12, 3]	0.684
11	Tumor.marker.CA=positive AND Fatigue = yes AND Total.Pelvic.Pain = Yes	Endometriosis.Stage=Stage4	[0, 1, 3, 13]	0.667
12	Prevoius.Cervical.Trauma = yes AND Dysmenorrhea = no AND Sport = yes	Endometriosis.Stage=Stage1	[14, 1, 2, 1]	0.682
13	Tumor.marker.CA=negative AND Abnormally of genital = Yes AND Prevoius.Cervical.Trauma = no	Endometriosis.Stage=Stage1	[11, 4, 0, 0]	0.632
14	Number of pregnancy ≥ 2.0 AND Duration of infertility ≥ 3.0 AND BMI ≥ 19.81	Endometriosis.Stage=Stage2	[1, 12, 1, 1]	0.684
15	Tumor.marker.CA=positive AND History of Galactorrhea = yes AND Weight ≥ 57.0	Endometriosis.Stage=Stage3	[0, 0, 10, 6]	0.550
16	Duration of infertility ≥ 11.0 AND Length of menstrual cycle ≤ 29.0 AND Dyspareunia = no	Endometriosis.Stage=Stage3	[0, 0, 13, 3]	0.700
17	Tumor.marker.CA=negative AND Menstrual Status≠iiregular AND Dysmenorrhea = no	Endometriosis.Stage=Stage2	[8, 18, 3, 1]	0.559
18	Pelvic.pain = yes AND Weight ≥ 66.0 AND L.endometriosisSymptoms≠endometrioma.cysts	Endometriosis.Stage=Stage1	[13, 1, 0, 2]	0.700
19	Abnormally of genital = Yes AND Length of menstrual cycle ≥ 28.0 AND Weight ≤ 66.0	Endometriosis.Stage=Stage2	[3, 14, 1, 1]	0.652
20	Menstrual Status=iiregular AND History of Galactorrhea = no AND Sport = no	Endometriosis.Stage=Stage1	[11, 0, 3, 1]	0.632
21	Tumor.marker.CA=negative AND Total.Pelvic.Pain = No AND History of Galactorrhea = no	Endometriosis.Stage=Stage2	[5, 12, 3, 0]	0.542
22	Prevoius.Cervical.Trauma = no AND L.endometriosisSymptoms≠endometriosis.lesions&endometrioma.cysts AND Height ≥ 157.0	Endometriosis.Stage=Stage1	[9, 6, 2, 0]	0.476
23	PelvicAndLowerBackPain = yes	Endometriosis.Stage=Stage4	[0, 4, 3, 8]	0.474
24	Number of abortion = yes AND Dyspareunia = yes AND Length of menstrual cycle ≥ 26.0	Endometriosis.Stage=Stage3	[5, 0, 8, 2]	0.474
25	Job≠House Wife AND Duration of infertility ≥ 4.0 AND Menarche ≤ 13.0	Endometriosis.Stage=Stage4	[0, 4, 4, 11]	0.522
26	TRUE	Endometriosis.Stage=Stage3	[162, 179, 237, 213]	0.299

### Model comparison and performance

**Fig. 8:**
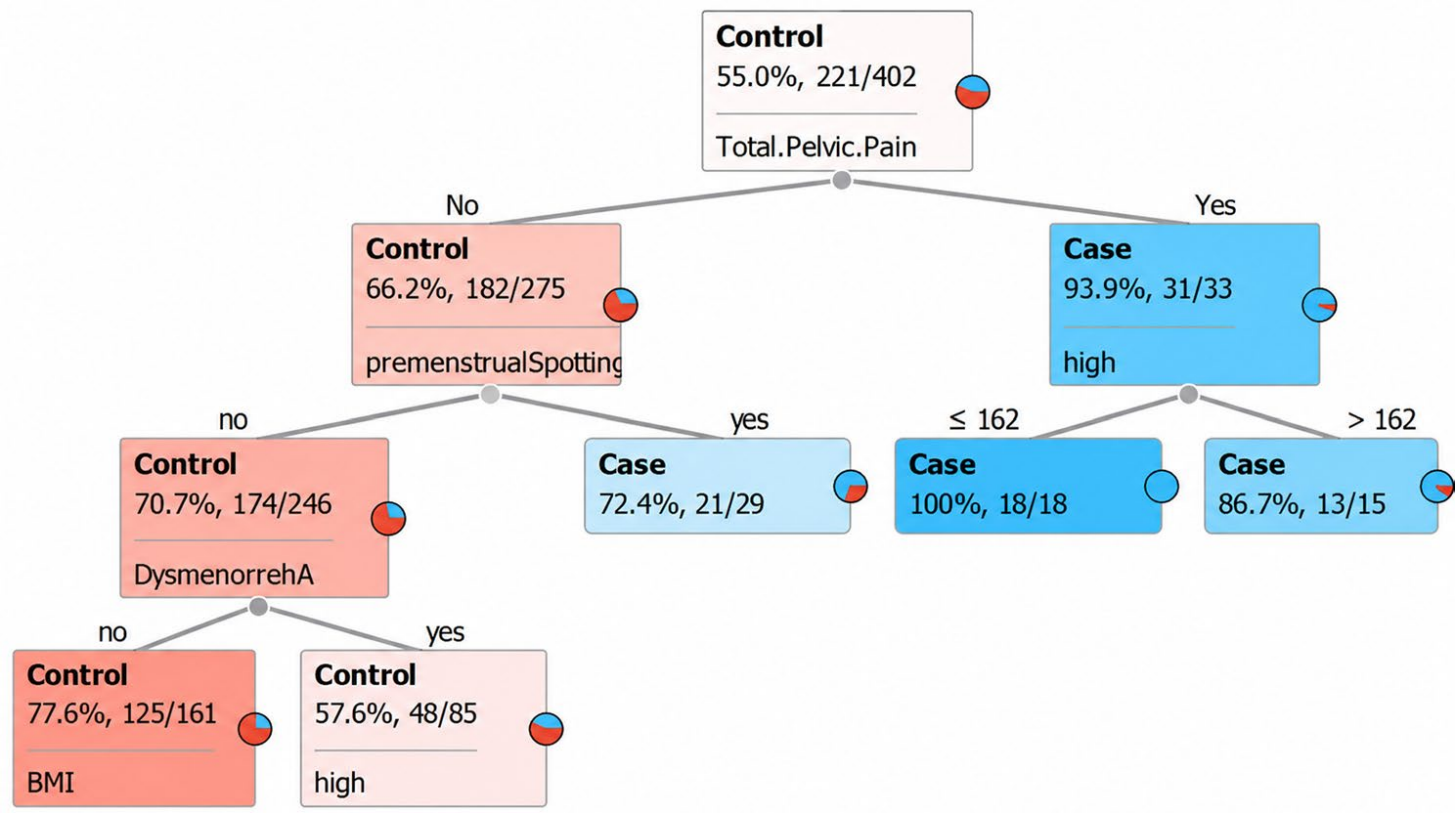
Decision tree for target class (control, case)

**Fig. 9:**
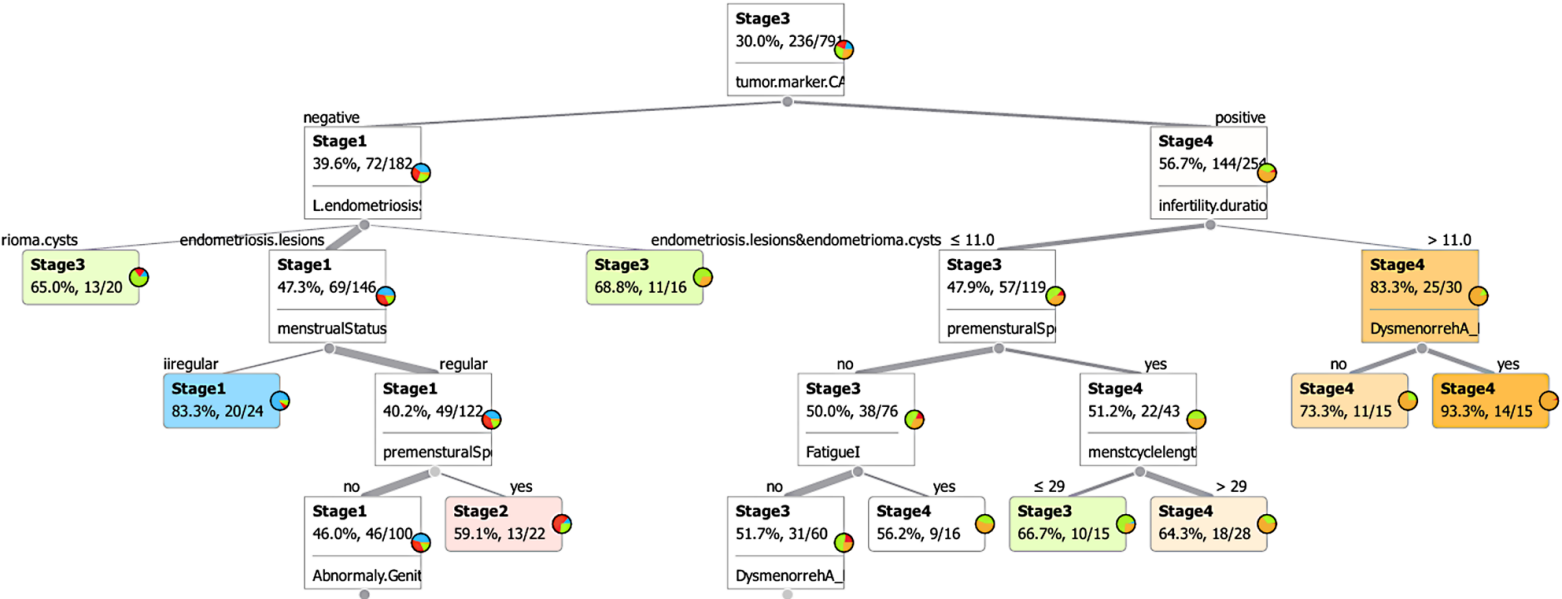
Decision tree for target class (Stages 1–4)

**Fig. 10:**
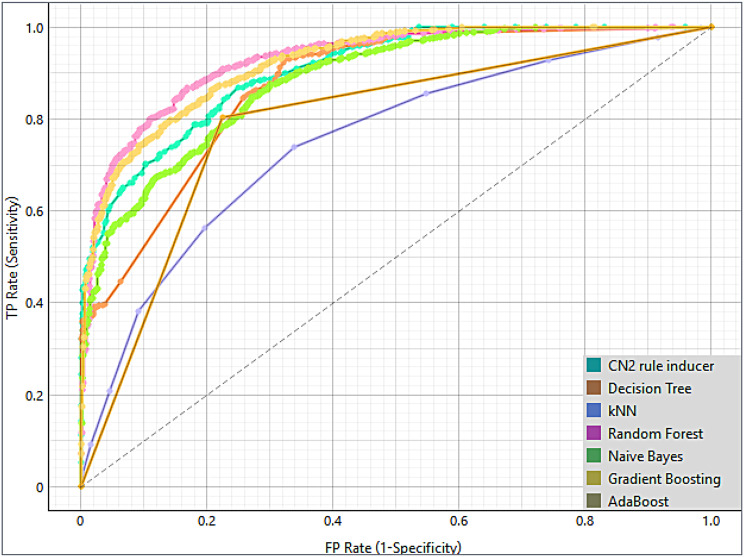
ROC for case group

**Fig. 11:**
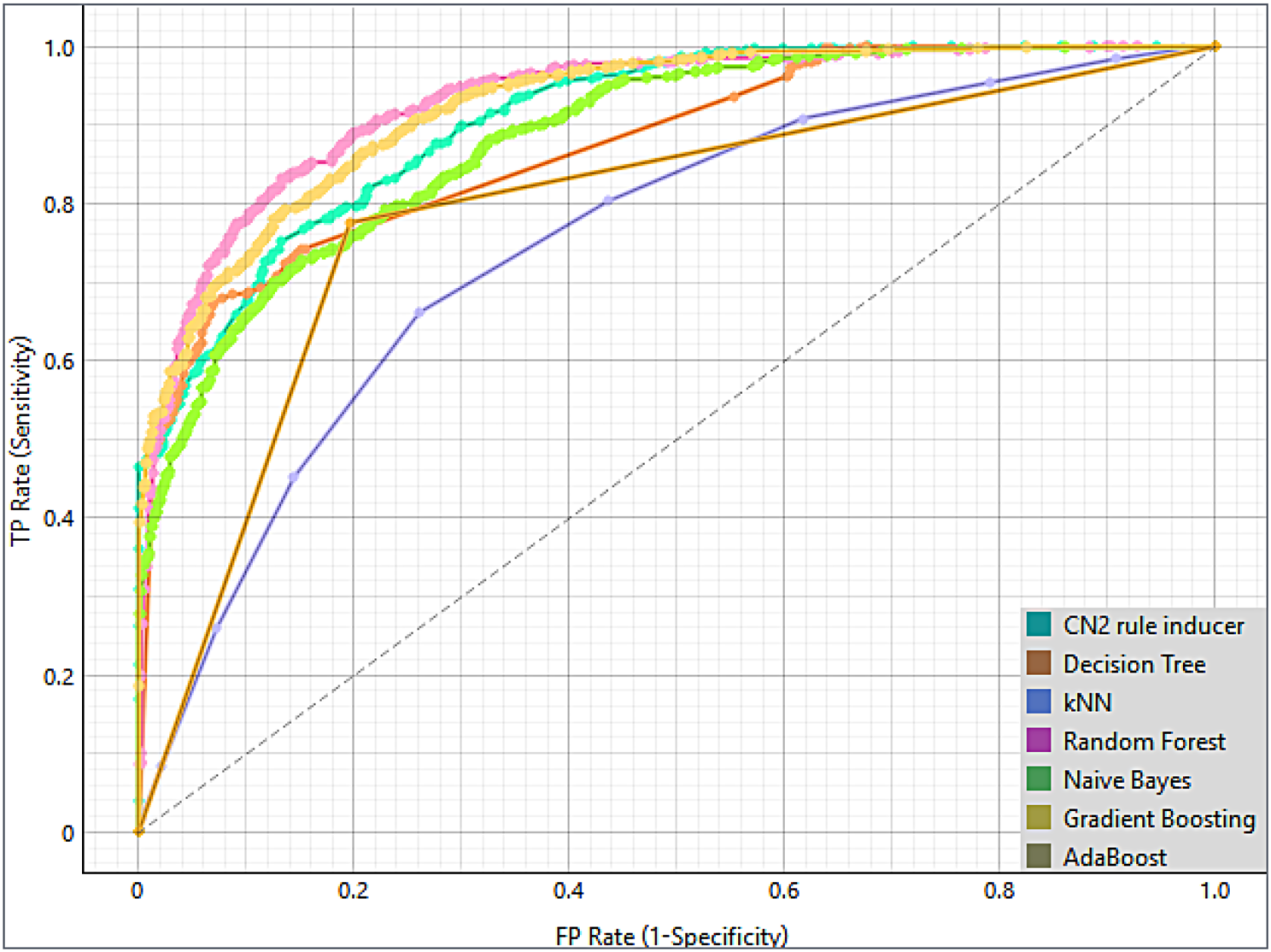
ROC for control group

We evaluated several classifiers for both the binary (case vs. control) and multi-class (stages 1–4) prediction tasks, using stratified 10-fold cross-validation. All machine learning models were trained with hyperparameter tuning, with parameters optimized through cross-validated grid search. A Decision Tree learner was implemented first, producing interpretable trees for case–control and stage classification (Figs. 8 and 9). The Decision Tree scored an AUC of 0.873 for case–control and 0.671 for staging. Decision Trees have the advantage of transparency and can handle discrete and continuous data with minimal preprocessing; their predictive accuracy was, however, surpassed by ensemble and rule-induction methods. As shown in Figs. 10 and 11, in the binary classification task, Gradient Boosting achieved the highest AUC of 0.931, whereas Random Forest achieved the highest CA (Classification Accuracy) of 0.845. The CN2 rule inducer had good and interpretable performance (AUC = 0.906, CA = 0.803), which was comparable to Gradient Boosting and Random Forest but with the additional advantage of comprehensible rules. Other binary results include: Naïve Bayes (AUC = 0.883, CA = 0.772), KNN (AUC = 0.750, CA = 0.702), and Adaptive Boosting (AUC = 0.790, CA = 0.790).

**Fig. 12:**
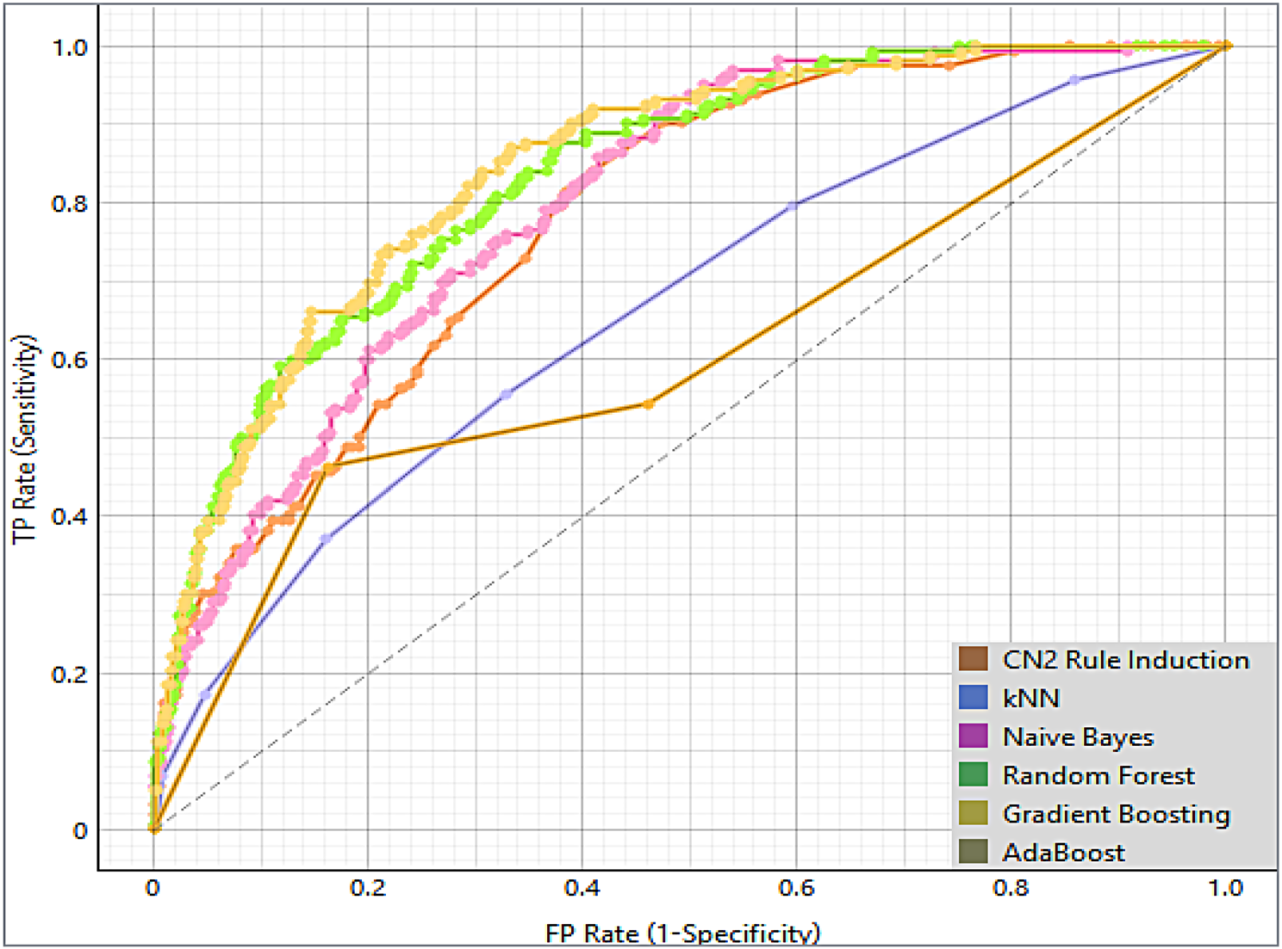
ROC for stage 1

**Fig. 13:**
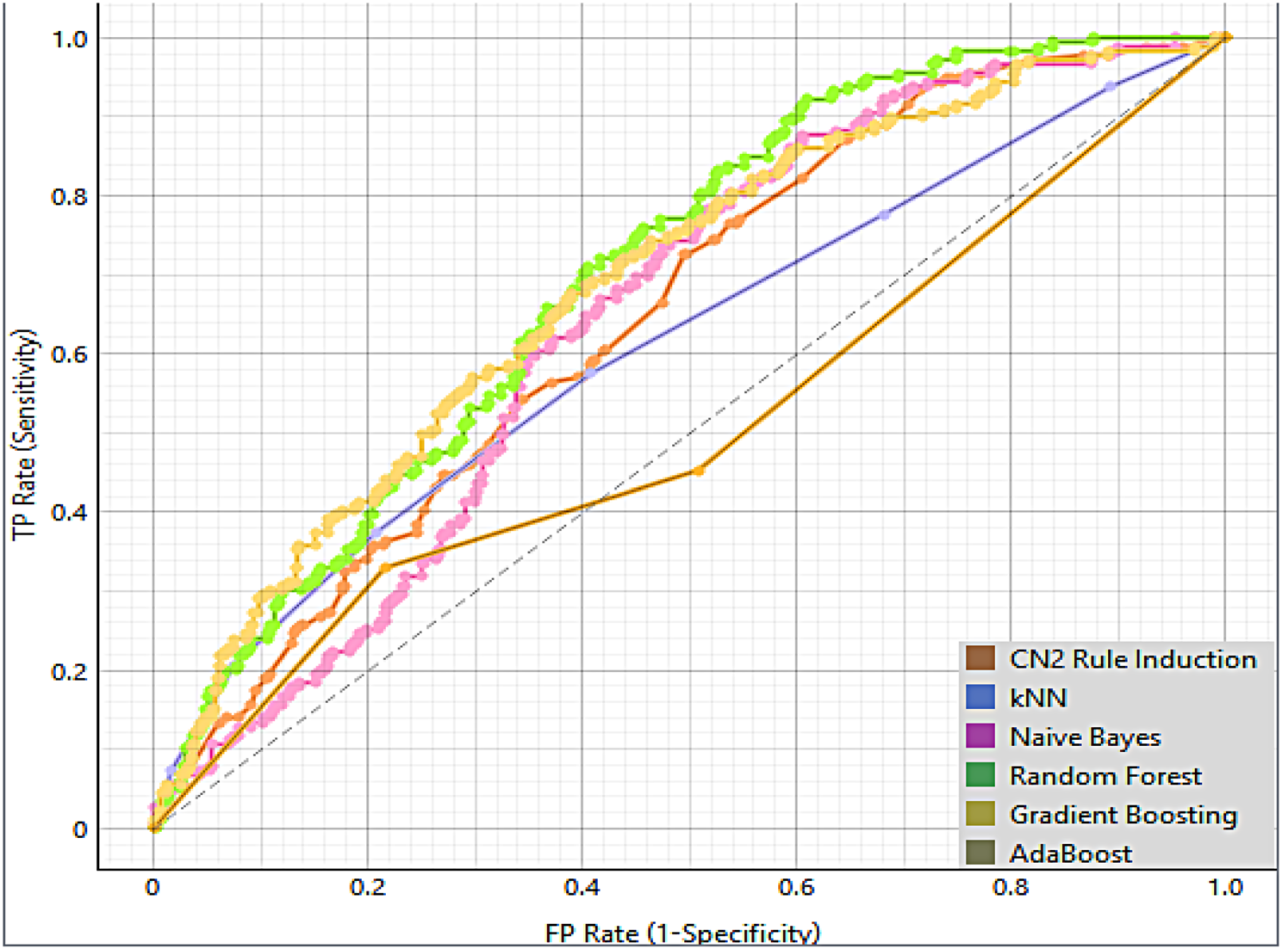
ROC for stage 2

**Fig. 14:**
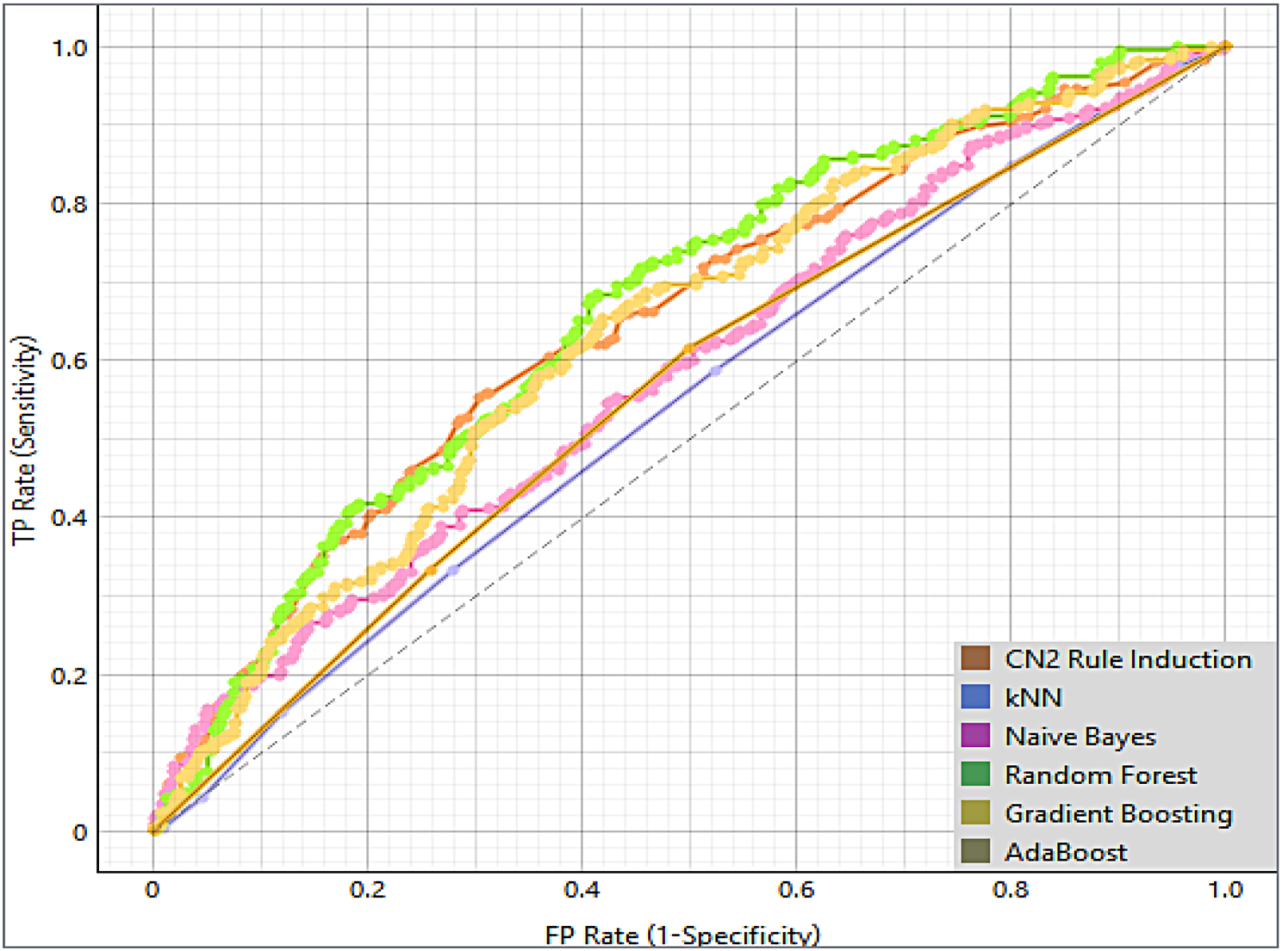
ROC for stage 3

**Fig. 15:**
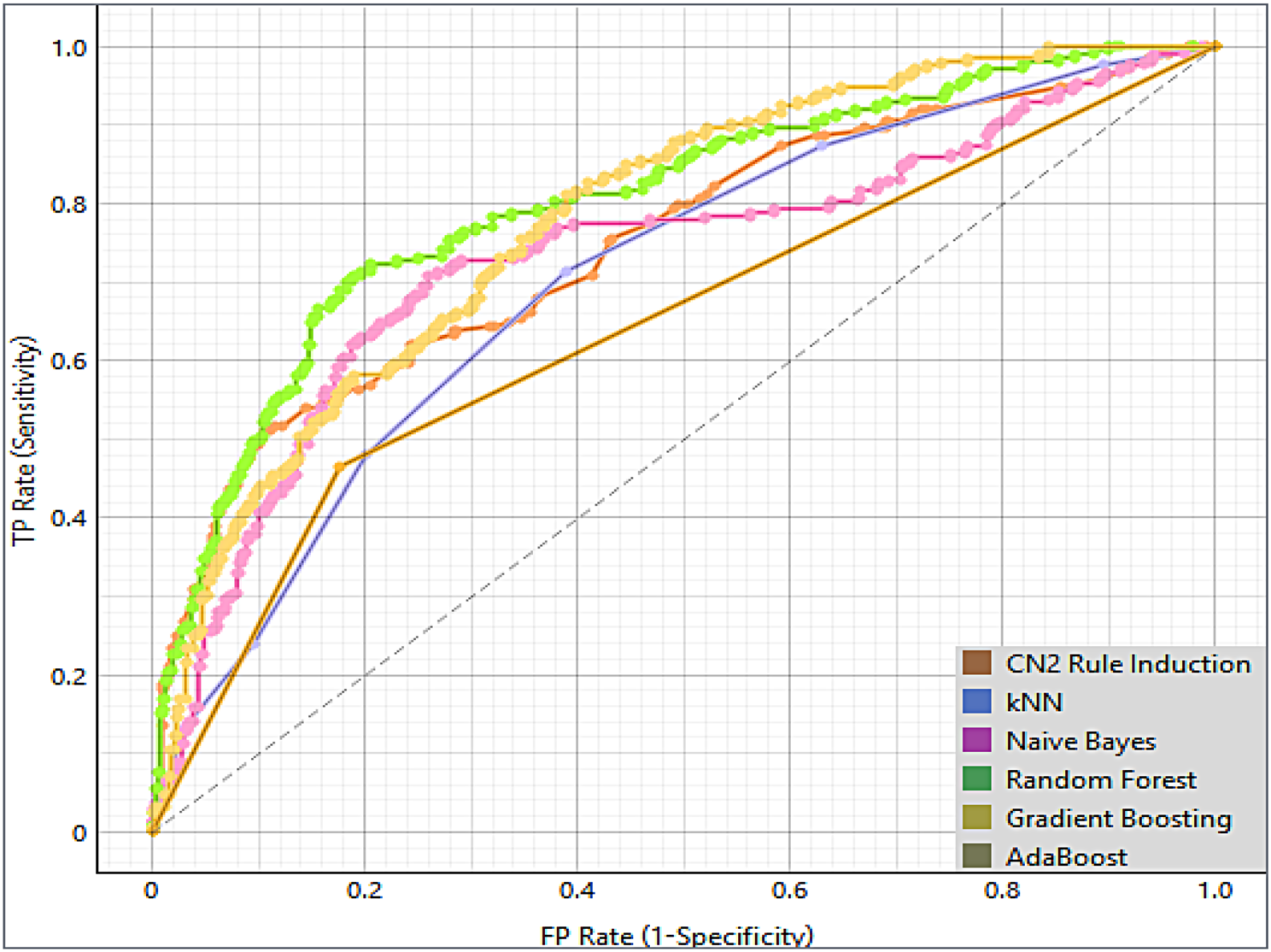
ROC for stage 4

For multi-class staging, performance declined as expected because of the complexity of the task. As shown in Figs. 12, 13 and 14, and 15, the best performance was obtained with Random Forest’s average AUC of 0.746, and Gradient Boosting’s AUC of 0.736, with CN2 ranking next with an AUC of 0.705. Decision Tree, Naïve Bayes, KNN, and Adaptive Boosting achieved lower staging performance. Tables 5 and 6 show the comparative results obtained from the CN2 rule inductions and other algorithms.

**Fig. 16:**
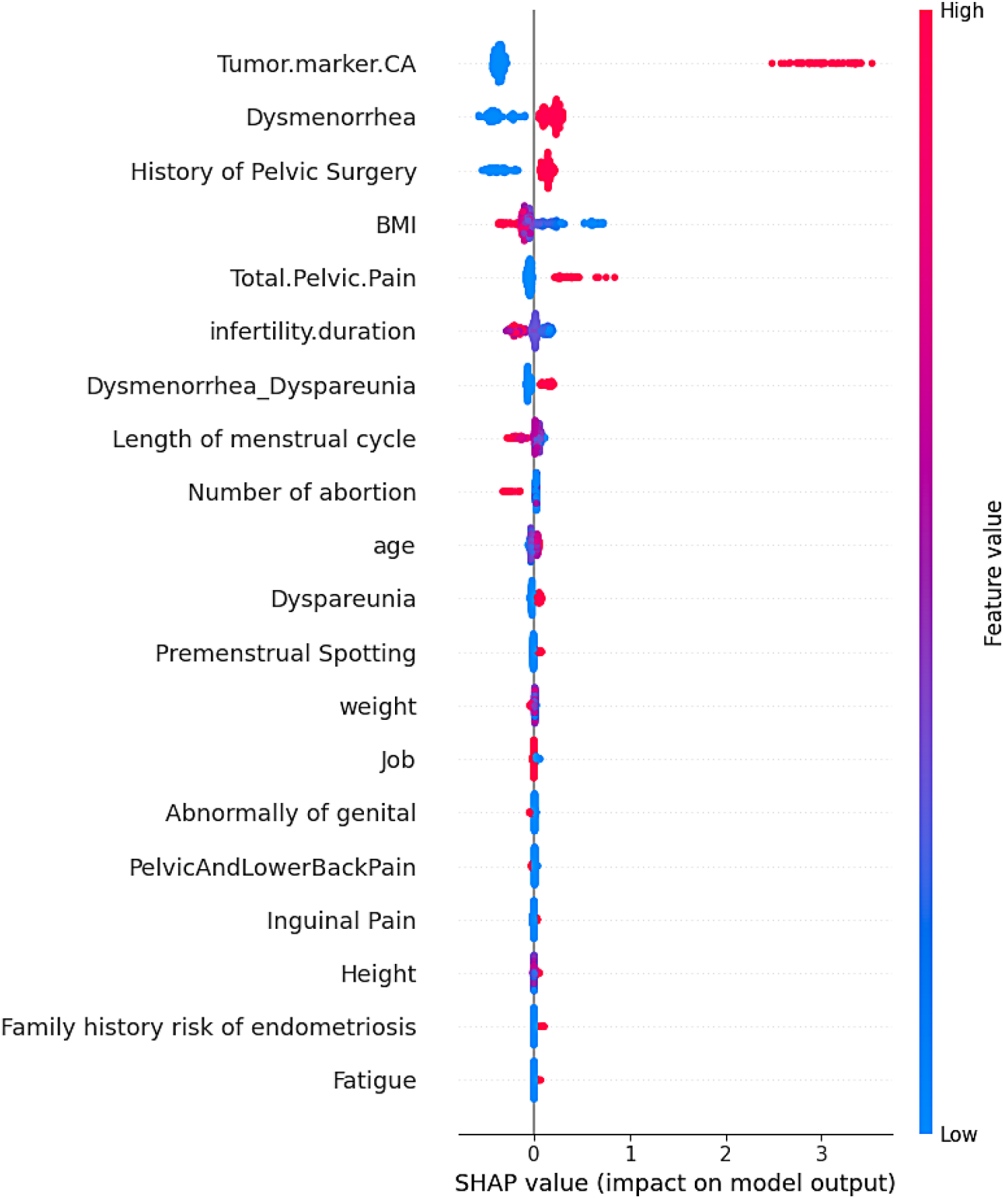
SHAP summary plot showing feature importance for case–control classification in endometriosis

**Fig. 17:**
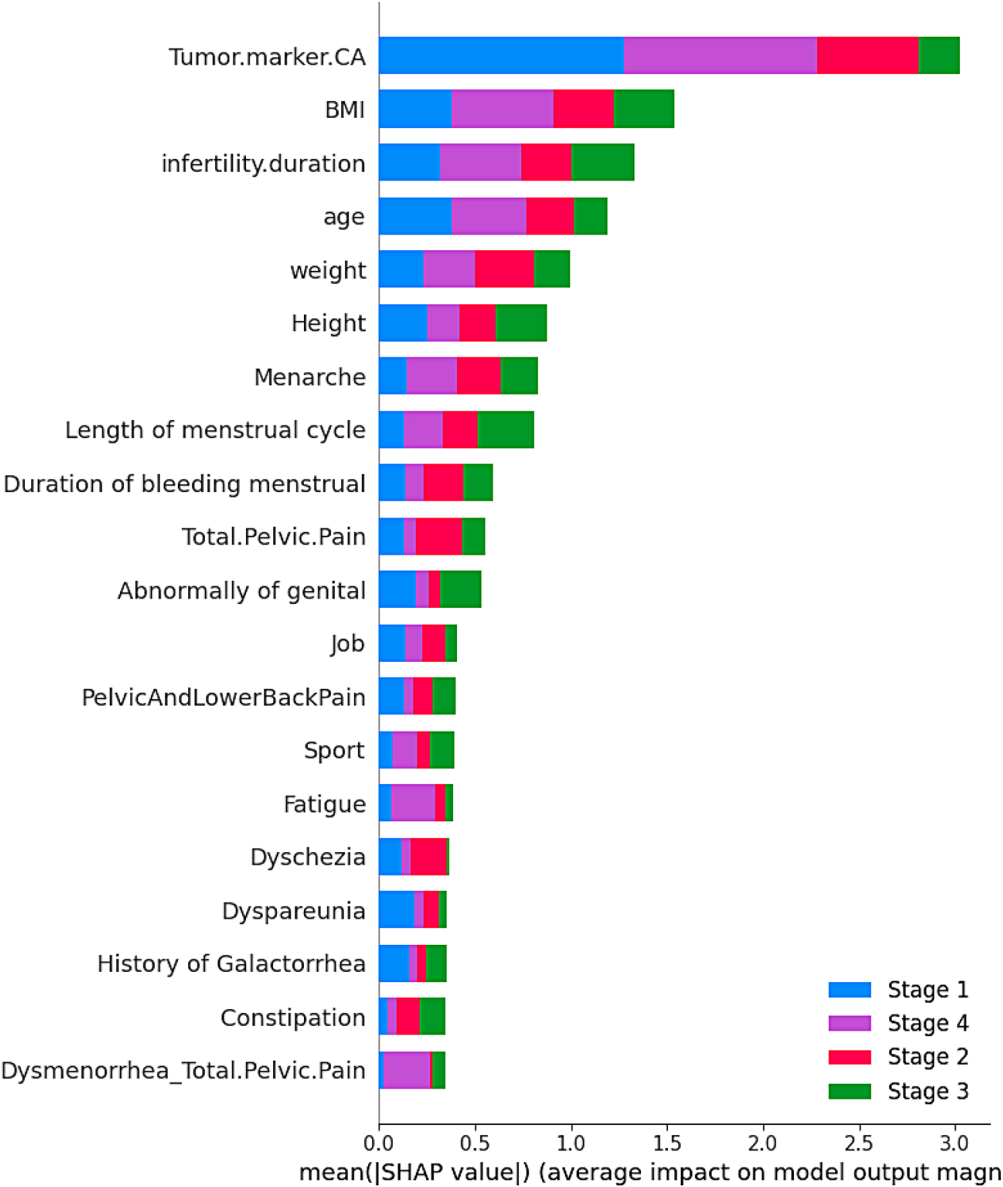
Mean SHAP values showing feature importance for endometriosis stage classification

As Gradient Boosting had given us the highest AUC amongst non-rule models, we utilized SHAP (SHapley Additive exPlanations) to interpret its predictions in comparison with CN2 rule induction model. SHAP assigns values to each feature for a specific prediction by computing how much the feature shifts the prediction from the baseline, providing an analysis of the model’s decision [37]. This is particularly important for clinical environments in which interpretability and trust are crucial [38]. As shown in Fig. 16, the horizontal axis represents the SHAP value for each feature: positive values push the prediction toward the endometriosis (Case) group, while negative values push it toward the Control group. The color of the points represents the actual feature value: blue indicates low values, red indicates high values, and purple indicates intermediate values. SHAP analysis showed that Tumor.marker. CA was by far the most influential feature, strongly pushing predictions towards the case class at high values (Positive). Additional top contributors included dysmenorrhea, history of pelvic surgery, BMI, total pelvic pain, infertility duration, and dyspareunia being influential. We also conducted SHAP analysis (Fig. 17) to explore feature effects by stages 1–4. Although Tumor.marker. CA was the most consistent driver of all stages, other features varied in importance by stage, BMI, duration of infertility, age, menarche, length of menstrual cycle, were the next most influential features, and symptom variables such as duration of bleeding menstrual, total pelvic pain, and genital abnormalities had variable influences by stage. The mean SHAP bar chart illustrates overall feature importance by stage.

Both Gradient Boosting and CN2 rule induction models identified the same influential factors, such as tumor marker CA, infertility duration, BMI, length of menstrual cycle, age, and pain-related symptoms such as dysmenorrhea, dyspareunia, and pelvic pain.

**Table 5:** Overall performance comparison of CN2 rule induction and other algorithms in case-control classification

Model	AUC	CA	F1	Precision	Recall	MCC
CN2 Rule Inducer	0.906	0.803	0.803	0.803	0.803	0.604
Decision Tree	0.873	0.797	0.796	0.798	0.797	0.593
Random Forest	0.924	0.845	0.845	0.845	0.845	0.689
KNN	0.750	0.702	0.702	0.702	0.702	0.402
Naïve Bayes	0.883	0.772	0.770	0.790	0.772	0.563
Adaptive Boosting	0.790	0.790	0.790	0.790	0.790	0.579
Gradient Boosting	0.931	0.840	0.840	0.840	0.840	0.679

**Table 6:** Overall performance comparison of CN2 rule induction and other algorithms in stages 1–4 classification

Model	AUC	CA	F1	Precision	Recall	MCC
CN2 Rule Inducer	0.705	0.445	0.445	0.446	0.445	0.255
Decision Tree	0.671	0.408	0.387	0.436	0.408	0.200
Random Forest	0.746	0.489	0.489	0.490	0.489	0.312
KNN	0.625	0.357	0.356	0.364	0.357	0.145
Naïve Bayes	0.685	0.405	0.381	0.417	0.405	0.213
Adaptive Boosting	0.580	0.394	0.395	0.396	0.394	0.190
Gradient Boosting	0.736	0.464	0.463	0.462	0.464	0.280

## Discussion

This study builds on past research by improving the understanding and diagnostics of endometriosis using a rule-based model, where the CN2 rule induction algorithm is applied to a comprehensive set of clinical data. The study, with a focus on all disease stages, was thus confronting gaps in already published literature, which very frequently narrow its focus to particular patient groups or disease stages. The CN2 algorithm was selected as it is able to generate comprehensible and interpretable rules that allow clinicians to understand key factors contributing to endometriosis and enable a clearer view in clinical decision-making. Our findings highlighted the potential of the CN2 model to reveal factors with significant influence on various stages of endometriosis. The model’s performance measures were strong, given the high AUC for distinguishing cases from controls and moderate performance in endometriosis staging. Certain key features, including Pelvic pain, dysmenorrhea, dyspareunia, age, severe bleeding, tumor markers, reproductive history, and BMI, are indicated by the induced rules as significant clinical features reflecting the diagnosis and staging of endometriosis. Such findings are comparable to state-of-the-art ML solutions. For example, a study achieved an AdaBoost symptom-based model with an AUC of 0.94 [20]. Another study achieved an AUC of 0.85 with CA125 and the neutrophil-to-lymphocyte ratio in a random forest [6]. Evaluated multiple ML classifiers on Iranian patients’ ultrasound and clinical data; one study used SVM, Random Forest, Extra-Trees, and Gradient Boosting, and all reached an AUC of 0.76 in their test cohort [27]. One study used a 21-item patient questionnaire with logistic regression. This approach achieved an AUC of 0.92 for identifying endometriosis [28]. Unlike opaque models, our rules clearly define important clinical variables in patient classification. This directly addresses “black box” AI concerns in endometriosis because interpretable approaches can more safely and confidently be used in practice [39]. Our findings combine data mining methods and clinical expertise. Overall, we achieve high accuracy while producing actionable clinical guidelines that clarify each predictor’s importance. Most of the variables that our rules have found to be influential are consistent with those found in the literature. Specifically, severe menstrual bleeding, tumor markers, dysmenorrhea, pelvic pain, and dyspareunia were all highly ranked features in the previous studies. For example, the top-performing ML model in one study ranked severe bleeding, dysmenorrhea, pelvic pain, and “dyspareunia” as among the highest-importance symptoms [20]. Similarly, one study applied XGBoost to a laparoscopic cohort and found that crampy pelvic pain and dysmenorrhea were among the strongest predictors of endometriosis [40]. Our rule that a positive CA-125 tumor marker indicates endometriosis is also consistent with research that reported that combining the neutrophil-to-lymphocyte ratio (NLR) with CA-125 substantially enhanced predictive accuracy [6]. Similarly, one study determined CA-125 and dysmenorrhea to be important characteristics for predicting stage 4 endometriosis [22]. We also noted that increased BMI favored a control (non‐endometriosis) classification. This association has previously been described in one study [20]. The rules presented by the CN2 model strongly align with established associations, supporting their credibility and reliability.

Many earlier models were trained on limited cohorts, focusing on only surgical patients or based on data available during late stages of diagnosis [22, 24, 41]. On the contrary, our analysis included women at different points of the disease and emphasized straightforward clinical and laboratory criteria. For example, models focusing on postoperative or imaging-confirmed cases may minimize nonspecific symptoms like pelvic pain [20], while our method uses available symptom data. Our multiclass stage classifier resulted in an average AUC of 0.705 for all stages, comparable to a study that reported an AUC of 0.744 for stage 4 endometriosis [22]. So, our results both confirm many published findings and extend them by providing a comprehensive, interpretable view across all patient stages.

The CN2-derived rules are readily applicable in the clinical setting. Every rule is an explicit combination of test results or symptoms that can be directly assessed by clinicians or embedded within decision‐support systems. For instance, a rule like pelvic pain and positive tumor marker CA would directly highlight the high risk of endometriosis. This aligns with the recent ESHRE guidelines, which emphasize the need for more thorough preoperative evaluation instead of immediate laparoscopy [42]. Importantly, using transparent rules may help reduce the well-documented diagnostic delay in endometriosis (often 6–12 years) by warning both providers and patients early on. These rules serve as a quick screen: patients who meet several risk rules might be triaged earlier for definitive testing. This fits within the larger promise of AI to reduce diagnosis time and costs [39]. Clinically, the induced rules imply more personalized patient management. Women who fulfill case criteria, for example, having severe dysmenorrhea with pelvic pain or irregular bleeding, could be prioritized for specialist evaluation or early treatment.

However, the performance of the CN2 model suffers from certain limitations regarding the classification of endometriosis stages. For staging classification, the performance of the model with an AUC of 0.705 indicates only moderate stage discrimination capability between stages 1–4. Performance would also be limited by the small number of patients for stages and the uneven stage distribution of the dataset. A dataset with higher informative content and more equally balanced with larger patient groups and more clinical features would probably enhance staging performance for the model and improve generalizability.

## Conclusion

The CN2 model provided interpretable rules that captured clinically relevant associations and identified important predictors in endometriosis, such as pelvic pain, severe bleeding, tumor marker CA, BMI, age, dyspareunia, dysmenorrhea, and reproductive history. These interpretable rules identified varying sets of features for each stage, providing a structured understanding of disease progression. In comparison to the recent literature, our findings validate known risk factors while extending them by providing an entire and interpretable framework for all stages of endometriosis. Clinically, our findings show the possibility of rule-based approaches to enable earlier detection, reduce delay in diagnosis, and enable more personalized patient management. Beyond endometriosis, the proposed approach can be applied to other conditions where interpretability is important for clinical decision-making. Future research should focus on improving the staging accuracy through larger and more diverse datasets, as well as the incorporation of additional clinical variables to improve both accuracy and generalizability of the model. Also, the rule-based model identified in this work can be adopted and used for other diseases that require the identification of influential clinical factors toward diagnosis and treatment.

## Data Availability

The data that support the findings of this study are not openly available due to sensitivity reasons and are available from the corresponding author upon reasonable request.

## Abbreviations

IUD: Intrauterine device
IUFD: Intrauterine fetal death
EP: Ectopic pregnancy
STD: Sexually transmitted diseases
PID: Pelvic inflammatory disease
BMI: Body mass index
SD: Standard deviation
ROC: Receiver operating characteristic
CA: Classification accuracy
F1: F1 Score
SHAP: SHapley Additive exPlanations
AUC: Area under the curve
MCC: Matthews correlation coefficient
KNN: K-nearest neighbors
